# Medicinal Chemistry Strategies for the Modification of Bioactive Natural Products

**DOI:** 10.3390/molecules29030689

**Published:** 2024-02-02

**Authors:** Yuyang Ding, Xiaoqian Xue

**Affiliations:** 1 Shenzhen Borui Pharmaceutical Technology Co., Ltd., Shenzhen 518055, China; yuyang_ding@hotmail.com; 2 Medi-X Pingshan, Southern University of Science and Technology, Shenzhen 518055, China

**Keywords:** natural bioactive compounds, medicinal chemistry, drug discovery, structure-activity relationships

## Abstract

Natural bioactive compounds are valuable resources for drug discovery due to their diverse and unique structures. However, these compounds often lack optimal drug-like properties. Therefore, structural optimization is a crucial step in the drug development process. By employing medicinal chemistry principles, targeted molecular operations can be applied to natural products while considering their size and complexity. Various strategies, including structural fragmentation, elimination of redundant atoms or groups, and exploration of structure-activity relationships, are utilized. Furthermore, improvements in physicochemical properties, chemical and metabolic stability, biophysical properties, and pharmacokinetic properties are sought after. This article provides a concise analysis of the process of modifying a few marketed drugs as illustrative examples.

## 1. Introduction

Natural bioactive compounds are structurally unique metabolites produced by a variety of organisms, including animals, plants, and microorganisms, that possess exceptional physiological activities. These compounds are valuable resources for the development of novel drugs, particularly in the ongoing battle against infectious diseases and cancer [1,2,3]. However, most natural bioactive compounds exhibit certain limitations in terms of their biological properties. These limitations include low activity, limited specificity, significant toxicity, and unfavorable pharmacokinetic profiles, which hinder their direct utilization as pharmaceutical agents. Therefore, it is imperative to optimize and modify their molecular structures to enhance their biological properties and achieve safety, efficacy, and control in drug applications.

Natural active compounds are highly regarded as valuable resources for discovering structurally innovative lead compounds. By modifying and optimizing the molecular structure, diverse libraries of compounds can be generated, harnessing the potential of natural product resources and bolstering the prospects of new drug development. The field of drug screening and development based on natural products has significantly advanced with the advent of bioinformatics technologies, including artificial intelligence (AI) and advanced computing. Techniques such as target prediction, metabolite profiling of natural products, and investigations into the dynamics and thermodynamics of pharmacophores have greatly facilitated the identification of lead compounds from natural sources. These approaches enable the exploration of structural transformations, commencing from the intact natural product, progressing to its fragments, and ultimately culminating in structural optimization [4,5,6].

## 2. Characteristics of Natural Products

Compared to chemically synthesized drugs, natural products are characterized by structural diversity and complexity, more chiral centers, fewer nitrogen and halogen atoms or aromatic rings, and other characteristics that will be discussed below.

### 2.1. Diversity and Complexity of Natural Product Structures

**Figure 1:**
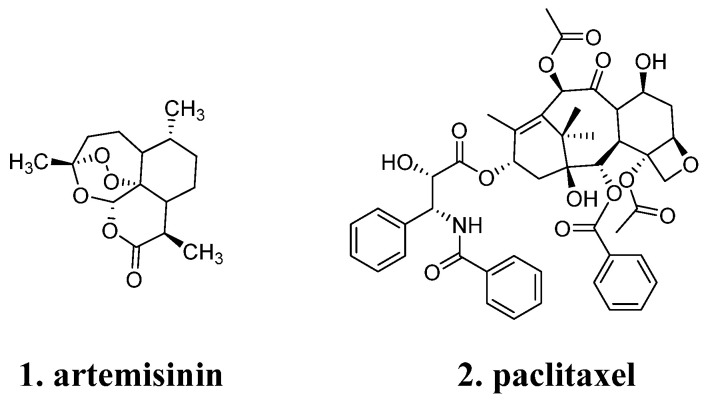
Structural diversity and complexity of natural products.

Natural products exhibit a remarkable diversity and complexity in their structures. Take **artemisinin** (**1**, Figure 1), for example, which contains a unique combination of a peroxide bond, lactone, and a bridged tricyclic system. These structural features not only preserve chemical reactivity but also ensure molecular stability [7]. Similarly, **paclitaxel** (**2**, Figure 1) possesses a fused tetracyclic framework with a 6-8-6-4 arrangement of functional groups, which contributes to its potent inhibition of microtubule proteins. However, the complex structures of natural products pose challenges in their chemical synthesis [8]. In some cases, natural product structures may contain “redundant” atoms that do not participate in target binding. This presence of redundant atoms can have negative implications on the physicochemical, pharmacokinetic, and pharmaceutical properties of the compounds. Therefore, it becomes crucial to remove these redundant atoms and fragments during the process of structural modification in order to enhance the efficiency of ligand binding [9].

### 2.2. High sp3 Carbon Content and Few Aromatic Rings

**Figure 2:**
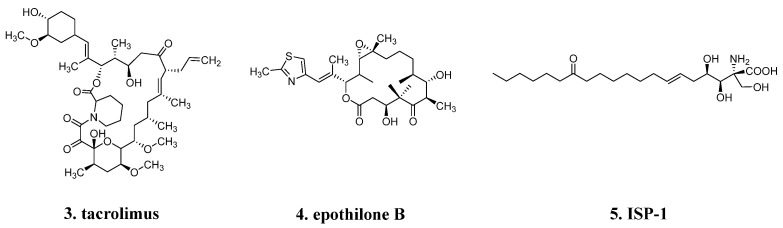
More aliphatic frameworks over aromatic rings in natural product.

Natural product structures often exhibit a high proportion of sp3-hybridized carbon atoms, which are commonly found in aliphatic chains or cyclic compounds. This unique feature imparts flexibility to these structures. For example, the immunosuppressant **tacrolimus** (**3**, Figure 2) and the antitumor agent **epothilone B** (**4**, Figure 2) possess large macrocyclic lactone structures that provide them with considerable flexibility. The active component **ISP-1** (**5**, Figure 2) in *Cordyceps militaris*, an immunomodulator, is a flexible linear compound.

Nature seems to prefer aliphatic rings over aromatic rings, as only 38% of known natural products contain aromatic systems [10]. Aromatic rings play a crucial role in interacting with drug targets through phenomena such as π-π stacking, hydrogen bonding, and van der Waals forces, thereby influencing pharmacological properties and bioactivity. Additionally, the introduction of various substituents or functional groups on aromatic rings can effectively modulate the properties, selectivity, and solubility of drug molecules [1,11].

### 2.3. Low Nitrogen and Halogen Content

Most natural products primarily consist of carbon, hydrogen, and oxygen, with a relatively low abundance of nitrogen atoms. When nitrogen atoms are present, their quantity is often limited. Nitrogen atoms can display nucleophilic characteristics and can exist in trivalent or pentavalent states. They can appear as basic salts or neutral amides, participate in ring formation, contribute to aromatization and fusion reactions, act as terminal or linking groups, and function as both hydrogen bond donors and acceptors. These properties enhance the binding efficiency between small molecules and ligands and can also have an impact on the drug’s solubility and bioavailability [12].

With the exception of bromine atoms commonly found in marine organisms, natural products generally contain a low amount of halogens [12]. Approximately 20% of small molecule anticancer lead compounds incorporate iodine, bromine, or chlorine. Halogens provide enhancements in lipophilicity and membrane permeability, and the electronegativity of halogens can augment the biological activity of the central molecule. For example, the inclusion of potent electron-withdrawing groups like fluorine can improve binding affinity, metabolic stability, physical properties, and selective activity [13,14,15].

### 2.4. Chirality and Stereochemistry

**Figure 3:**
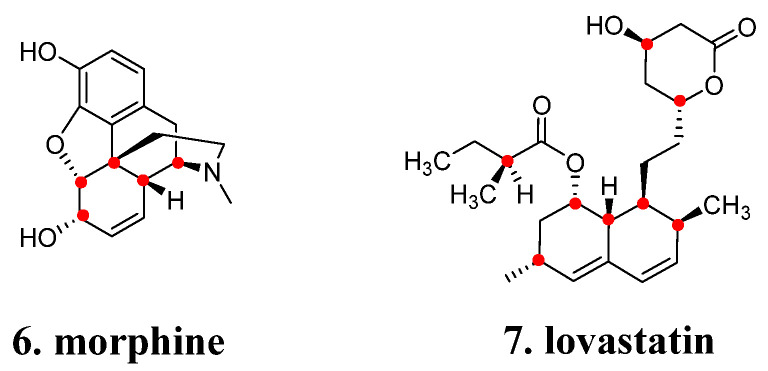
More chiral centers in natural products.

The generation of natural products involves a series of enzymatic reactions, wherein the stereospecificity of these reactions determines the stereochemical attributes of the resultant products, including chiral centers, axes, and cis-trans isomerism [12]. For instance, **morphine** (**6**, Figure 3) consists of 21 non-hydrogen atoms, forms five fused rings, and possesses five chiral centers (red dots). **Lovastatin** (**7**, Figure 3), on the other hand, comprises 28 non-hydrogen atoms, has eight chiral centers (red dots), and two conjugated trans double bonds. Dealing with chirality and stereochemistry can be as challenging as handling complex structures. Consequently, during chemical synthesis, it is advisable to minimize unnecessary chiral elements while maintaining activity and pharmacokinetic properties [1,11].

## 3. Employ Structure-Based Drug Design to Natural Products Optimization

**Figure 4:**
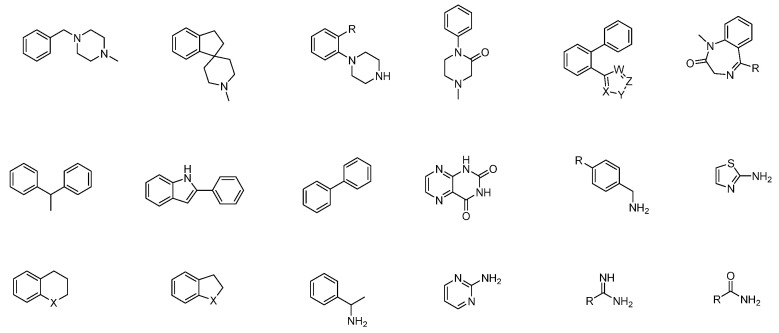
Examples of privileged fragments.

The aforementioned characteristics present a range of options for structural modification and transformation, allowing for novel discoveries in research and development. Compared with complex natural drugs, the structure of chemical drug molecules is relatively simple, with a higher content of aromatic rings, especially many nitrogen-containing aromatic heterocycles, resulting in a higher overall nitrogen content in the molecule. This design can form additional hydrogen bond donors and acceptors. The linking mode of cyclic systems is relatively simple, and it is often linked by short linkers such as amide bonds or methylene. Some structural fragments frequently appear in multiple drug molecules and have specific structures and functions, which are closely related to the activity and characteristics of drugs (Figure 4).

Privileged fragments (Figure 4) refer to small molecular fragments or scaffolds that are highly represented in bioactive compounds and exhibit a wide range of biological activities. In medicinal chemistry, they have several advantages [16]: (1) Activity and affinity: The utilization of privileged fragments in multiple drugs is primarily driven by their demonstrable higher activity and affinity, facilitating specific interactions with biological macromolecules, such as proteins and enzymes. These fragments have exhibited favorable biological activities in several drug compounds, thereby enhancing the probability of uncovering pharmaceutically active compounds; (2) Highly optimized properties: given the recurrent presence of privileged fragments in multiple pharmaceuticals, their structural and functional characteristics have undergone rigorous validation and verification within an extensive repertoire of drug design and optimization endeavors. Consequently, these fragments have garnered considerable attention and refinement, offering distinct advantages in enhancing pharmacokinetic properties, pharmacological profiles, and selectivity of drug candidates [17]; (3) Flexibility in structural modification: privileged fragments assume a pivotal role as core scaffolds in medicinal agents, providing ample opportunities for subsequent structural modifications to fine-tune their specific characteristics. This inherent flexibility enables chemists to personalize these fragments by manipulating side chains and incorporating additional functional groups, fostering improved optimal drug performances [18]; (4) Rich structural diversity: although privileged fragments possess predetermined skeletal frameworks, their adeptness for modifications at diverse positional and orientational geometries engenders a wealth of structural diversity [19]. In doing so, privileged fragments facilitate the integration of molecular diversity, effectively expanding the scope of medicinal chemistry investigations to encompass numerous potential targets and pathways. In conclusion, privileged fragments exhibit high activity and affinity, and, through optimization and research, they have demonstrated significant advantages in medicinal chemistry.

**Figure 5:**
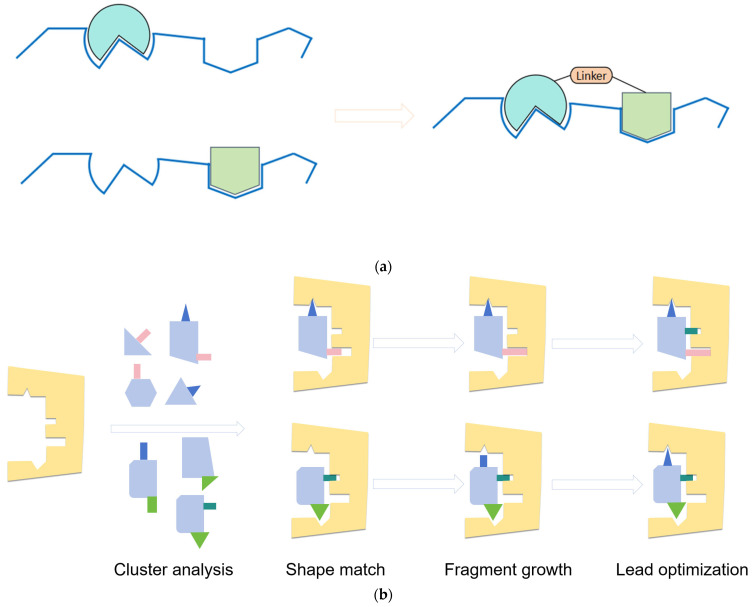
Constructing and optimizing strategies for molecular structure in medicinal chemistry. (**a**) Fragments linking schematic; (**b**) Scaffold-based drug discovery in a schematic illustration.

Due to their controllable structure, privileged fragments offer relative simplicity in synthesis compared to complex natural compounds, allowing for further optimization through structural modifications. This flexibility enables chemists to personalize these fragments by adjusting side chains, introducing additional functional groups, and establishing abundant structure-activity relationships, ultimately resulting in rich structural diversity (Figure 5). This facilitates the attainment of improved drug performance [20].

In medicinal chemistry, “simplifying complexity” is an important remodeling strategy. It involves simplifying the structural core of complex active natural products, partially or completely transforming them into privileged scaffold structures that are easier to synthesize and possess stronger pharmacological effects. Additionally, it can involve deconstructing active natural products into smaller molecular fragments, reassembling and optimizing the entire new scaffold using fragment-based drug design principles, and further modifying it through local structural modifications to enhance its activity and pharmacological efficacy.

Over the past 20 years, with the widespread application of high-throughput screening techniques and computer-aided drug design, as well as the increasing availability of protein structure databases and natural product databases, a foundation has been established for structure-based remodeling of active natural products. By integrating techniques such as virtual screening, high-throughput screening, large databases, structural biology information, and computational chemistry, it is possible to employ structure-based drug design strategies such as scaffold hopping and privileged scaffold replacement to optimize certain active natural lead compounds.

## 4. Modification Strategies of Successful Examples

### 4.1. From ISP-1 to Siponimod

**Figure 6:**
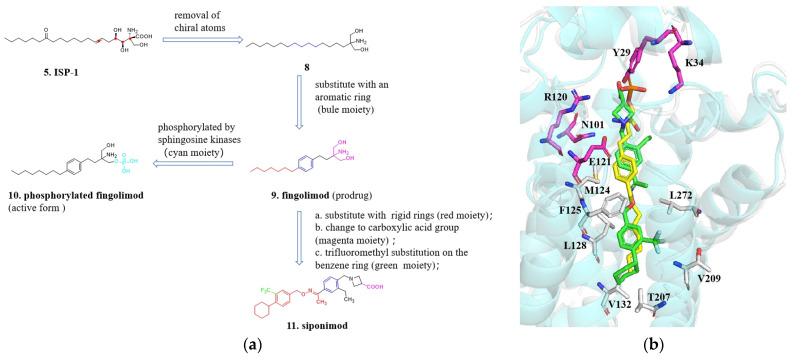
(**a**) The modification strategies from ISP-1 to siponimod; (**b**) Superimposed crystal structures of **fingolimod** (yellow) and **siponimod** (green) in S1PR1. **Fingolimod** and **siponimod** exhibit linear conformation in the pocket, the polar headgroup of molecules form potential polar interactions with the sidechains of Y29, K34, N101, R120 and E121 (magenta). The nonpolar tailgroup of **siponimod** inserts into a deeply hydrophobic cavity of M124, F125, L128, V132, T207, V209 and L272 (gray).

The ultimate goal of modifying natural products is to develop active compounds into medicines. The transformation process from **ISP-1** to **siponimod** (**11**, Figure 6a) serves as an example of natural product modification. The strategies and methods used in this process have significant implications for modifying other natural compounds (Figure 6a).

**ISP-1** is a natural product and it exhibits immunomodulatory effects by acting as a receptor for sphingosine-1-phosphate [21]. I**SP-1** possesses a complex structure with three chiral centers, a trans double bond, and both an amino group and a carboxyl group. However, due to its high toxicity and low solubility, I**SP-1** cannot be used as a medication without further modifications or adaptations [22]. To simplify the structure, reduce or eliminate chiral centers, improve activity, and enhance pharmacokinetics, compound **8** was selected as a lead compound for structural modifications. Through a series of transformations and optimizations, **fingolimod** (**9**, Figure 6a) was eventually developed. **Fingolimod** is a symmetrical molecule that has undergone modifications such as removal of the ketone group, trans double bond, and chiral carbons. This molecule lacks chiral and stereoisomeric factors and incorporates a benzene ring into the long chain, which reduces the number of saturated carbons and facilitates synthesis and conformational rigidity. **Fingolimod** was introduced to the market in 2010 for the treatment of multiple sclerosis [23,24,25,26,27]. The success of **fingolimod** can be attributed to the substitution of the alkyl chain with an aromatic ring. Alkyl chains, being flexible in nature, can exist in various conformations, which may not favor the attainment of a “high concentration” of active conformations. Hence, incorporating factors that restrict conformational flexibility in the chain, such as replacing a portion of the saturated carbon chain with a phenyl ring, can offer advantages in terms of potency, pharmacokinetics, safety, and physicochemical properties [22,25,26]. **Fingolimod** functions as a prodrug that is transformed into its active form by sphingosine kinase 2 in the liver after oral absorption. In light of this, a new compound called **phosphorylated fingolimod** (**10**, Figure 6a) was designed [28]. In the subsequent stages of development, the researchers discovered **Siponimod**, a novel S1P1 receptor agonist that was developed by Novartis. This compound was designed by replacing the flexible lipid chain of **fingolimod** with a rigid aromatic ring and cyclohexane, and by introducing a trifluoromethyl group onto the aromatic ring. These structural modifications were implemented to enhance the selectivity of **siponimod** in its interaction with specific receptors, thereby potentially influencing its pharmacological activity [29]. In initial structure-activity studies, the trifluoromethyl group on the benzene ring was found to significantly impact activity, boosting it by over 30 times compared to the unsubstituted hydrogen atom. 

Despite the structural differences between **siponimod** and **fingolimod**, they share similar molecular sizes and pharmacophore features, and their distribution patterns exhibit resemblances (Figure 6b). In terms of potency, **siponimod** exhibits an EC_50_ value of 0.4 nM, while its EC_50_ for the S1P3 receptor, where its activity is undesirable, is at 5 μM, thus demonstrating a high level of selectivity. Furthermore, studies conducted on monkeys have revealed an oral bioavailability of 71% for **siponimod**, with a plasma half-life (T_1/2_) of 19 h [30]. From **ISP-1** to **Siponimod**, the transformation process involves simplifying the complex structure of a natural product with multiple chiral centers and a long flexible chain, which has poor pharmacological properties, into a small molecule chemical drug with a clearly defined drug-like structure. Several transformative strategies can be summarized as follows: (1) In the initial stage of remodeling, the structure is simplified by removing chiral centers as much as possible to reduce the difficulties in chemical synthesis. This allows the synthesis of a controllable drug scaffold for systematic structure-activity relationship studies; (2) For the long flexible liner alkyl chain, the advantage of introducing a pharmaceutically strong aromatic core is that it increases molecular rigidity, and the aromatic ring structure enables further structural modifications; (3) The flexible tail of **Fingolimod** is further replaced with a benzene ring and a cyclohexane while maintaining its hydrophobic properties. This not only enhances rigidity but also allows for additional modifications by introducing functional groups onto the benzene ring; (4) It can be rationalized using molecular modeling based upon solved S1P1crystal structure to do the optimization (PDB: 3V2Y). Based on the predicted binding mode, it appears that the carboxylic acid headgroup of **siponimod** is able to form salt bridges with Lys34 and Arg120 and a hydrogen bond with Tyr29. These electrostatic interactions are strong and serve to anchor the ligand molecule in its binding pocket [31]. 

Although there is a significant difference in structure from **ISP-1** to **Siponimod**, the remodeling process follows the principles of simplification and advantageous fragment replacement, leading to the generation of simplified lead compounds. Through structure-activity relationship studies, guided by structure-based drug design principles, and with the assistance of structural biology, the structure is further elaborated. These strategies demonstrate an effective approach for the transformation of active natural products by combining scaffold hopping, privileged fragment replacement, and guidance from structural biology information.

### 4.2. The “Statin” Drugs

**Figure 7:**
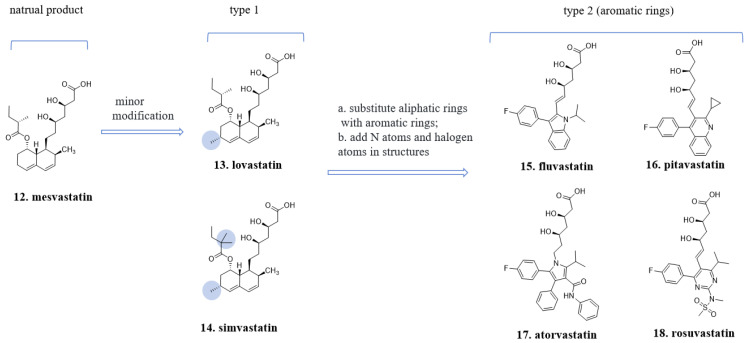
Structural optimization from **mesvastatin** to type 1 and type 2 “statin” drugs.

**Mesvastatin** (**12**, Figure 7), which is isolated from the fermentation broth of the fungus *Penicillium citrinum*, was the first inhibitor discovered that targets hydroxymethylglutaryl-coenzyme A (HMG-CoA) reductase, the rate-limiting enzyme in cholesterol synthesis in the body. The chemical structure of **lovastatin** (**13**, Figure 7) includes eight chiral carbons, with two within the upper lactone ring and six on the lower hexahydronaphthalene ring. It was introduced to the market in 1987 as a medication for lowering cholesterol levels [32]. The lactone ring, formed by a dihydroxy acid, is an important pharmacophore feature, while the hexahydronaphthalene structure serves as a backbone and hydrophobic fragment crucial for enzyme binding, although the presence of chiral centers is not necessary. 

Subsequent “statin” drugs that entered the market retained the dihydroxy acid structure but underwent significant changes in the lower part of the molecule, which lacks chiral centers. Enzyme binding is primarily governed by hydrophobic-hydrophobic interactions. For instance, **fluvastatin** (**15**, Figure 7), **pitavastatin** (**16**, Figure 7), **atorvastatin** (**17**, Figure 7), and **rosuvastatin** (**18**, Figure 7) all share the same spatial orientation of the two hydroxyl groups in the dihydroxy acid fragment, but their structural backbones are transformed into indole, quinoline, pyrrole, and pyridine rings, respectively. These structural variations contribute to their lipid-lowering effects [33].

**Figure 8:**
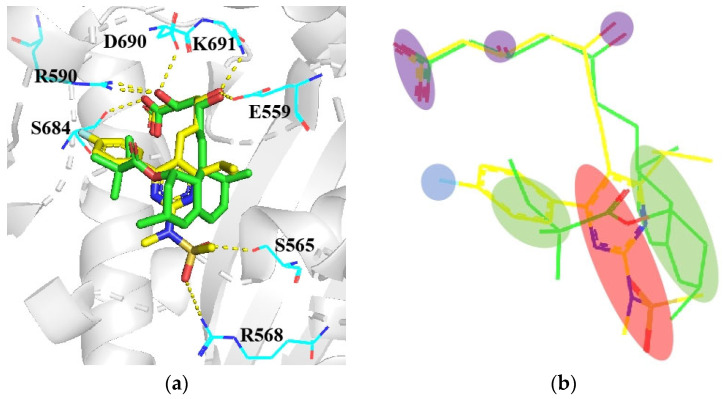
Comparing the differences between type 1 and type 2 by analyzing the structures and binding modes of **simvastain** (yellow, PDB:1HW9) and **rosuvastain** (green, PDB:1HW1). (**a**) Mode of binding of **simvastatin** and **rosuvastain**. R590, S684, D690, K691, and E559 are similar for all inhibitors and are indicated by the yellow dotted lines. Additional interactions between R590 and the fluorophenyl group are present in the **rosuvastatin**, it also forms hydrogen bonds between S565, R568, and sulfone oxygen atoms (red dot lines); (**b**) the Pharmacophore model of **simvastain** and **rosuvastain**.

Interestingly, despite the different structural types of synthetic statin drugs, they share similar binding modes. Figure 8a illustrates the interactions of simvastatin and rosuvastatin with the amino acid residues. Compared to simvastatin, rosuvastatin utilizes aromatic rings with halogen and nitrogen atoms to replace the hexahydronaphthalene ring [34]. The additional fluorophenyl motif and nitrogen atoms enhance the binding affinity between rosuvastatin and the target protein (Figure 8b) [35].

The spatial arrangement and structure of compounds is critical for their biological activity. However, the construction of complex stereogenic structures in synthetic medicinal molecules differs fundamentally from the formation of stereoisomers in natural products. Stereogenic structures in natural products are formed via complex biosynthetic pathways, relying on several endogenous reactions and selective enzymes. In contrast, medicinal chemists can construct and control the stereogenic structure of synthetic molecules via chemical synthesis and purification techniques. Therefore, the methods for constructing stereogenic structures of synthetic medicinal molecules are fundamentally different from the way in which natural products form stereoisomers. The stereogenic structure of natural medicinal molecules is often constructed with unique stereochemistry through chiral sp3-hybridized carbon atoms or complex ring systems that happen to achieve binding specificity with proteins, generating biological activity. For example, the hexahydronaphthalene structure of type 1 “statin” drugs is formed by five chiral carbon atoms with sp3 hybridization, creating a unique stereoconfiguration of fused rings. It is difficult to synthesize analogs of this structure or make modifications or substitutions on this structure. It is also challenging to conduct comprehensive structure-activity relationship studies on this ring system. In type 2 molecules, the transformation of the hexahydronaphthalene structure into privileged structures such as quinolines and indoles proceeds by substituting the ester group with a benzene ring and incorporating a fluorine atom onto the benzene ring. Additionally, the methyl group can be substituted with a propyl or cyclopropyl group as desired [36,37].

Regarding the four aromatic ring systems of Atorvastatin, its spatial configuration is achieved by connecting different aromatic rings and adjusting the dihedral angles between the rings, forming a unique stereostructure. Fragments are connected either through direct aromatic ring connections or by using amide bonds as linkers, which is a common connection method in the synthesis of drug molecules. Many classic organic named reactions, such as the Suzuki reaction, Buchwald–Hartwig reaction, Ullmann reaction, and others, efficiently enable the coupling between aromatic rings [37,38,39]. Compared to the ester bond connection commonly found in natural products, the amide bond exhibits higher stability and inertness, making it relatively stable under changes in temperature and pH. It is less susceptible to acid-base hydrolysis and therefore exhibits good stability in biological systems. The stereoelectronic and donor-acceptor interactions of the amide bond can modulate the pharmacokinetic properties of the drug. The incorporation of amide bonds into the structure of drug molecules can improve their lipophilicity and hydrophilicity, allowing for better control of their absorption, distribution, and metabolic behavior. In the structure of rosuvastatin, more nitrogen atoms are introduced. Nitrogen atoms are typically capable of acting as hydrogen bond acceptors or donors. Due to their electronegative nature, nitrogen atoms are ideal candidates for forming hydrogen bonds with positively charged hydrogen atoms. Hydrogen bonding is one of the key ways in which drug molecules interact with biomacromolecules such as receptors or enzymes. It not only influences the activity, specificity, and affinity of the drug, but also alters its physicochemical and metabolic properties. Compared to atorvastatin, rosuvastatin displays enhanced hydrophilicity, resulting in reduced penetration of the blood-brain barrier. This characteristic prevents central nervous system stimulation and does not interfere with a patient’s sleep [40,41].

In summary, privileged fragments concatenation are powerful techniques for constructing active drug skeletons and accelerating the synthesis and optimization of drug molecules. These methods allow for the replacement of natural scaffolds with controllable synthetic structures. These advantageous structures often consist of standardized chemical building blocks, enabling efficient creation of active compound libraries with similar structural features [42]. The coupling of aromatic fragments is commonly used in chemical synthesis because it offers a fast and highly selective method for constructing versatile scaffolds with high yields. Aromatic pharmacophores are particularly suitable for substitution and fragment growth, especially when combined with small molecule-protein crystallography information [36,43]. This combination enables precise adjustments in fragment growth and substitution, making it easier to explore structure-activity relationships. The substitution and modification have the potential to enhance therapeutic efficacy and reduce adverse reactions.

### 4.3. From Phloridzin to Dapagliflozin

**Figure 9:**
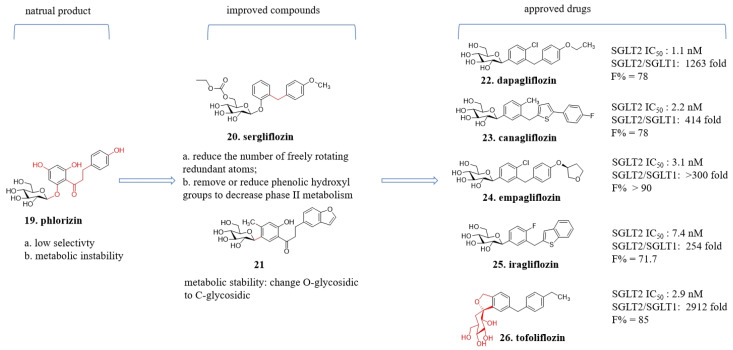
Structural optimization from bioactive natural product to approved drugs.

For 150 years, **phlorizin** (**19**, Figure 9), a phloroglucinol glucoside, has been known to be present in the roots, stems, and fruit peels of fruit trees. Extensive research has been conducted to explore its potential as a medicinal agent and pharmacological tool. It has been discovered that **phlorizin** exerts a hypoglycemic effect by inhibiting the sodium-glucose co-transporter 2 (SGLT2) in the renal tubules, leading to the excretion of glucose in the urine and reductions in blood glucose levels. However, **phlorizin** also inhibits the sodium-glucose co-transporter 1 (SGLT1) in the intestinal mucosa, which limits its use as a drug and may cause side effects [44,45]. Nevertheless, **phlorizin** serves as a valuable lead compound for further research. Structural modifications have been made to achieve the following objectives: (1) Elimination of the inhibitory effect on SGLT1 while improving selective inhibition of SGLT2; (2) Reduction or removal of phenolic hydroxyl groups to decrease phase II metabolism and prolong in vivo retention time; (3) Enhancement of the in vivo stability of compound glycosidic bonds [46,47].

Between two aromatic rings of dihydrochalcone, there are four rotatable bonds. Reducing the number of single bonds can be beneficial in maintaining an active conformation and enhancing the activity [46,47,48]. Through a series of explorations, it was discovered that the benzene rings could be connected by methylene groups. Compared to **phlorizin**, the structure of **sergliflozin** (**20**, Figure 9) has reduced the number of freely rotating redundant atoms and increased the rigidity of the molecular framework, thereby enhancing the selectivity of the scaffold. However, **sergliflozin** still has stability problems and is not available as a drug [49].

The glycosyl group is a pivotal pharmacophore; however, the O-glycosidic linkage exhibits poor metabolic stability as it is susceptible to cleavage mediated by β-glucosidase enzymes. Through a series of explorations, compound **21** was disclosed, which was characterized with C-aryl glucosides and meta-substituted diarylmethanes. This transformation sustained its activity and selectivity while bolstering its metabolic stability. Starting with compound **21** (Figure 9), a series of structure-activity relationship studies were conducted. Among the C-aryl glucosides compounds, **dapagliflozin** (**22**, Figure 9) demonstrated exceptional stability and selectivity. It presented IC_50_ values for SGLT2 and SGLT1 at 1.1 and 1390 nmol·L^−1^, respectively, augmenting its selectivity more than a thousand-fold. Jointly developed by BMS and AstraZeneca, it advanced to Phase III clinical trials. It earned approval from the European Union in 2012, marking its debut as the inaugural SGLT2-targeting drug for type 2 diabetes [50]. Simultaneously, a series of SGLT2 inhibitors were launched, such as **canangliflozin** (**23**, Figure 9), **empagliflozin** (**24**, Figure 9), and **ipragliflozin** (**25**, Figure 9). These were independently developed in different companies. Those molecules almost have the same pharmacophore features and similar scaffolds [51,52,53]. **Tofoliflozin** (**26**, Figure 9) is a non-glucosides SGLT2 inhibitor and possess high selectivity and good absorption characteristics [54].

**Figure 10:**
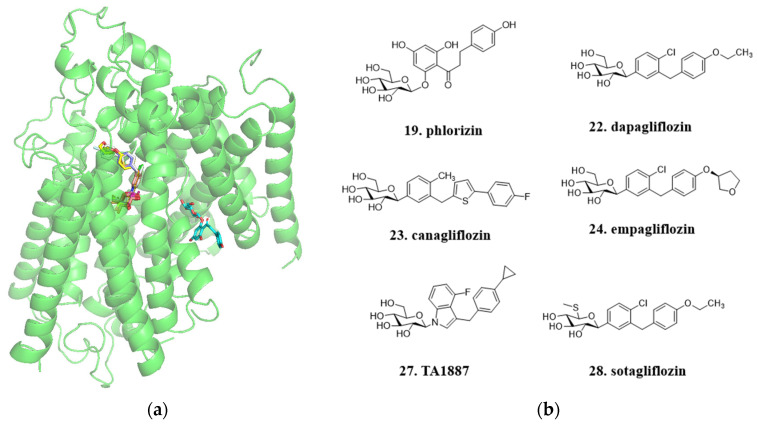
**Phlorizin** has a different binding site compared to other SGLT2 inhibitors. (**a**) Mode of binding of **phlorizin** (blue), **TA1887** (**27**), **dapagliflozin**, **sotagliflozin** (**28**), **canangliflozin**, and **empagliflozin** to SGLT2, (PDB ID: 7VSI, 8HDH, 8HIN, V8HB0, 8HEZ, and 8HG7); (**b**) The chemical structures of related compounds.

During the early stages of development, due to the lack of a crystalline complex between phlorizin and the protein, the specific binding mode between the target protein and the compound was not well understood. Researchers primarily relied on traditional medicinal chemistry optimization methods such as scaffold hopping and structure-activity relationship studies to guide compound modifications. Although the detailed interaction details between phlorizin and the target protein could not be directly elucidated, researchers were still able to achieve excellent therapeutic effects through compound improvements. However, since 2022, several reports have emerged regarding the crystalline complexes between SGLT2 protein and small molecules, revealing the binding mode of these compounds with the protein [55]. From the latest small molecule-protein co-crystallization analysis, despite the structural similarities between the natural compound phlorizin and the marketed drugs, they are actually not located in the same active pocket (Figure 10a). This work provides a framework for understanding the mechanism of the SGLT2 inhibitors and also develops a foundation for the future rational design and optimization of new inhibitors targeting these transporters.

**Figure 11:**
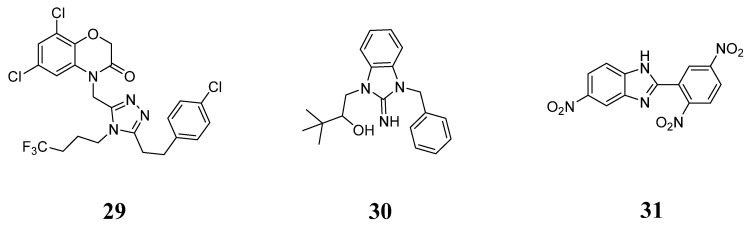
Non-glycosides SGLT2 inhibitors.

This finding provides new directions and opportunities for further research and enables scientists to explore non-glycoside SGLT2 inhibitors with different structural scaffolds. While glycoside-based SGLT2 inhibitors derived from root extracts have shown promising efficacy in medical practice, the development of non-glycoside SGLT2 inhibitors remains of significant scientific and commercial value. Compounds **29**, **30**, and **31** (Figure 11) were identified through a ligand-based virtual screening strategy, combined with pharmacophore models and structural clustering analysis, as structurally novel non-glycoside SGLT2 inhibitors [56,57,58]. The exploration of the structure optimization and structure-activity relationship of compounds **29**, **30**, and **31** is currently underway, and more researchers are expected to contribute to the development of novel SGLT2 inhibitors in the future.

In summary, these advanced technologies significantly enhance the speed and efficiency of drug development. Advanced structural biology techniques such as cryo-electron microscopy can be used to determine the co-crystal structures of natural compounds and proteins, thereby identifying their binding sites, binding strength, binding modes, and effects on organisms. This contributes to our understanding of the mechanisms of action of active molecules in vivo and provides guidance for drug design and discovery. For compounds or targets that are difficult to co-crystallize, the alphafold technology can be utilized to predict the binding modes of small molecules with proteins. While there may be some deviation between the predicted results and the actual binding state, it still provides relevant information for each stage of modifying active natural products.

### 4.4. Structure Simplification

Structure simplification serves as a strategic approach to optimize natural products. By reducing the complexity of natural product structures while maintaining or decreasing their activity, it makes them easier to synthesize and facilitates comprehensive exploration of structure-activity relationships for further studies.

**Figure 12:**
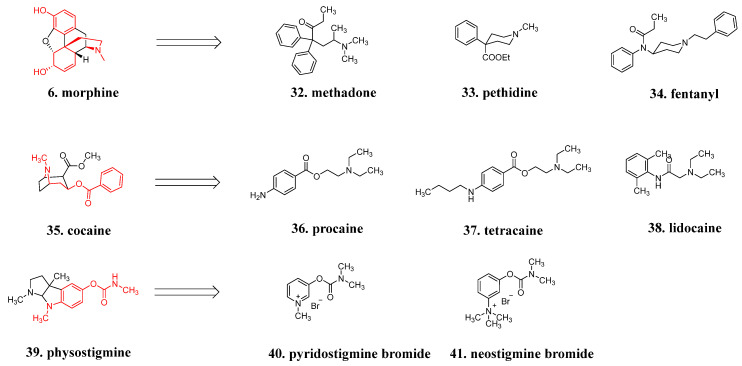
Simplification strategies for natural products.

Take morphine, for example: none of the five chiral centers in morphine are essential for binding with opioid receptors. Although **methadone** (**32**, Figure 12) and **pethidine** (**33**, Figure 12) contain one chiral carbon, there is no difference in activity between their enantiomers. **Fentanyl** (**34**, Figure 12) is a symmetrical molecule without chirality. Compared to the complex skeleton of morphine, fentanyl is simpler and easier to synthesize. The elimination of chiral centers and stereochemical configurations, which has made it feasible to explore a series of investigations on structure-activity relationships, solubility, absorption, and metabolism, is characteristic of this class of drugs with a simplified framework [59]. Similarly, the cyclic structure of alkaloid **cocaine** (**35**, Figure 12) is cleaved, simplifying its structure and allowing for the synthesis of non-chiral local anesthetics like **procaine** (**36**, Figure 12), **tetracaine** (**37**, Figure 12), and **lidocaine** (**38**, Figure 12) [60]. **Physostigmine** (**39**, Figure 12) is a parasympathomimetic alkaloid and a reversible cholinesterase inhibitor. Due to its chemical instability in the body, modified compounds such as pyridostigmine **bromide** (**40**, Figure 12) and neostigmine **bromide** (**41**, Figure 12) have been developed. Unlike physostigmine, which is a tertiary amine, these synthetic quaternary ammonium salts have limited penetration into the central nervous system, resulting in a lower likelihood of adverse effects such as orthostatic hypotension. However, they still effectively improve muscle tone in patients with myasthenia gravis [12].

## 5. Optimization of Bioactive Natural Products Using Computer-Guided and Structure-Based Screening Strategies

**Figure 13:**
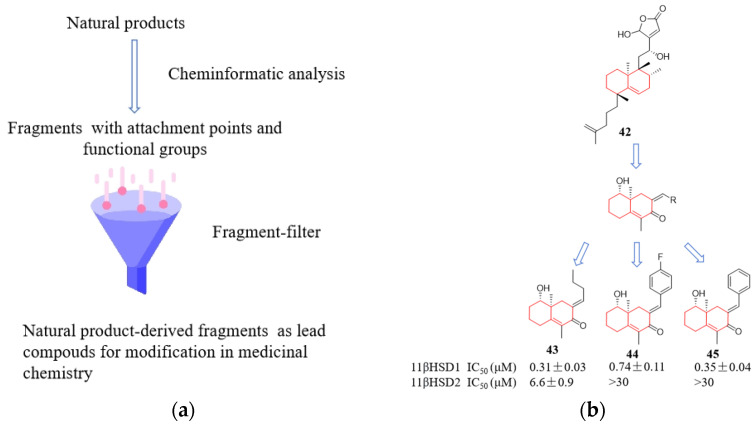
Natural products and their computationally generated fragments as inspiration for drug discovery. (**a**) Virtual natural product fragmentation; (**b**) The 2-(benzyloxy)naphthalene core derived from naphtalenic lignan lactone was utilized for designing inhibitors of 5-lipoxygenase.

The scaffolds derived from natural products can be dissected into simpler and more easily synthesized fragment-like scaffolds either manually or computationally [61]. These scaffolds inherit distinct conformational and physicochemical features from the original natural product templates, making them suitable for exploring chemically relevant space with biological activity. The general design strategy follows a flow-process diagram, involving the gradual simplification of the structural complexity of the parent compound (natural product) into virtual fragments, resulting in the formation of small and chemically appealing scaffolds (Figure 13a). Natural products have provided inspiration, while computer programs have enhanced the efficiency of rational molecular design. Koch et al. designed a compound library from the marine product dibromo-dysidiolide, where 19% of the compounds showed activity as inhibitors of 11β-hydroxysteroid dehydrogenase type I (Figure 13b) [10,61]. Similarly, adopting the concept of extracting scaffolds, Wetzel released the Scaffold Hunter software (https://scaffoldhunter.sourceforge.net) in 2009, which employs deconvolution analysis of structurally complex natural products to obtain virtual skeletal trees, thus making the chemical structure data of complex bioactive substances more intuitive [62,63]. The structural classification of natural products (SCONP) is an organizing principle for charting the known chemical space explored by nature. SCONP arranges the scaffolds of the natural products in a tree-like fashion and provides a viable analysis and hypothesis-generating tool for the design of natural product-derived compound collections [61,64]. Compared to high-throughput screening, this approach has a higher hit rate. However, the activity of compounds obtained through molecular design based on natural product fragments often ranges from weak to moderate, making subsequent structural optimization an indispensable step in improving activity.

The discovery of early natural active drugs occurred before a complete understanding of disease mechanisms, and researchers initially relied on animal pathological models for drug screening. Later, with advancements in molecular pathology, studies began to focus on specific enzymes or proteins as targets for drug screening. Due to the development of computer technology, bioinformatics, and structural genomics, an increasing number of important protein targets and their crystal structures, such as adrenergic receptors [65], potassium ion channels [66], and sodium-calcium exchangers [67], have been resolved. This has made it possible to use crystallographic structures for the screening and design of natural active compounds.

**Figure 14:**
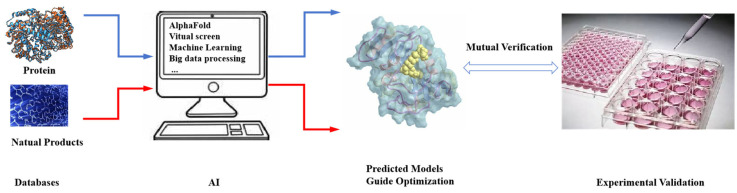
Employ AI in natural products optimization.

Unlike traditional high-throughput screening methods based on cells and enzymes, virtual screening based on protein structures can greatly shorten the time and reduce the cost of obtaining active natural products [68,69]. After obtaining the active compounds through virtual screening, further activity experiments can be conducted to verify the results. Combining computational simulations with experimental results, especially the mutual verification of structure-activity relationships and computational simulation results, can guide the next step of drug design (Figure 14). With the clear structure of drug target proteins, computer simulations can be used to simulate the binding of drug molecules to target proteins, and even obtain corresponding complex crystal structures directly. This provides information for the targeted modification of drug molecules. Therefore, virtual screening based on protein structures and the resolution of complex crystal structures provide a faster and more effective way to obtain and optimize active natural compounds.

Currently, the main structural biology techniques for obtaining protein target structures include X-ray crystallography [70,71], nuclear magnetic resonance (NMR) spectroscopy [72,73], and cryo-electron microscopy (cryo-EM) three-dimensional reconstruction [74,75,76]. However, these methods may not be applicable to some complex proteins, which limits the study of many important proteins and drug design. To overcome this limitation, DeepMind has developed a protein structure prediction software based on neural networks, known as Alphafold (https://alphafold.com). Alphafold uses large-scale protein structure databases for training and can accurately predict protein folding, secondary structure, domain contacts, and other information. This technology can quickly and accurately predict protein structures, which is very useful for drug design and virtual screening [77,78,79,80]. By predicting protein structures, researchers can use computer simulations to predict the interaction between drug molecules and target proteins, and thus predict the inhibitory activity, affinity, and selectivity of drugs. This method can accelerate the drug development process and provide more targeted drug design strategies. Alphafold’s high accuracy in protein structure prediction has demonstrated its advantages. However, for some complex proteins or protein complexes, there are still challenges that need to be further improved and developed.

Protein structure databases can also provide references for drug design and virtual screening, helping scientists quickly find drug candidates related to specific targets. There are multiple protein structure databases worldwide; PDB was established by the Brookhaven National Laboratory in the United States in 1971. It is a molecular structure database and an open-source database that is currently maintained by the Research Collaboration for Structural Bioinformatics (RCSB). It is considered the most important database in the field of structural biology [81]. The majority of its data comes from experimentally determined three-dimensional structures of biomolecules, including proteins, as well as some nucleic acids, sugars, and complexes formed by nucleic acids and proteins. In the drug screening process, the quantity and quality of compounds in the library are crucial. These compounds need to be representative and be able to cover as many active chemical skeleton types as possible. Some common natural product databases are shown in Table 1, and some of these databases overlap with each other. The following introduces several well-known databases.

The initial compounds obtained from virtual screening are subsets of the compound databases used in the screening process and require further experimental validation, such as compound activity and binding affinity to target proteins. Molecular docking prediction involves estimating the binding free energy, i.e., the binding affinity, between small molecule compounds and target proteins. However, for enzymes and receptors, predicting binding affinity alone is insufficient, and more specific experiments are needed to determine whether the small molecule compounds act as inhibitors (antagonists) or activators. The following are several techniques that directly measure the binding affinity between small molecules and target proteins. For example, microscale thermophoresis (MST), isothermal titration calorimetry (ITC), and surface plasmon resonance (SPR). MST measures the strength and rate of binding between small molecule compounds and target proteins using a microscale heat gradient [86,87]. ITC determines the binding constants and thermodynamic parameters by measuring the heat released or absorbed during the interaction between small molecule compounds and target proteins [88,89]. SPR quantifies the binding affinity and kinetic parameters by monitoring changes in reflected light caused by the binding of small molecule compounds to target proteins [90].

## 6. Brief Remarks

Natural products are secondary metabolites produced by organisms for their own growth and propagation, rather than being specifically designed for the treatment of human diseases. However, due to their inherent activity and limitations in terms of pharmaceuticals, structural modifications are necessary. These modifications should be personalized and tailored to address the specific properties and limitations of the natural products under study, with the aim of optimizing therapeutic efficacy, pharmacokinetics, safety, and biopharmaceutical characteristics. Looking at successful examples of natural products evolving into drugs, the extent of structural changes can vary from drastic transformations to minor alterations involving only a few atoms or functional groups. While there is no fixed pattern, the underlying principles and concepts of structural modification remain consistent. Refining their structures through these modifications aims to enhance activity potency and selectivity. Modern technologies such as cryo-electron microscopy (cryo-EM), AlphaFold, and natural compound databases have revolutionized the field of drug discovery and development, offering exciting new possibilities for modifying bioactive natural products. By utilizing these technologies and resources, researchers can use structure-based drug design in medicinal chemistry to optimize bioactive natural products and improve various aspects of their properties, ultimately developing safer and more effective natural drugs.

## Figures and Tables

**Table 1:** Commonly used databases of natural products.

Dataset	Number of Compounds	Description	Web Site	Ref
ZINC	224,205	A free database of commercially-available compounds for virtual screening	https://zinc15.docking.org/substances/subsets/natural-products/ (accessed on 26 January 2024)	[82]
Super Natural III	790,096	A freely available database of natural products and natural product-based derivatives. Information on pathways, mechanism of action, toxicity, vendor information.	https://bioinf-applied.charite.de/supernatural_3/index.php?site=home (accessed on 26 January 2024)	[83]
TCM Database	37,170	It is currently the world largest and most comprehensive free down small molecular database on traditional Chinese medicine for virtual screening.	http://ismart.cmu.edu.tw (accessed on 26 January 2024)	[84,85]
CMNPD(Marine Natural Product Database)	32,000	CMNPD is a manually curated open access knowledge base dedicated to marine natural products research. It provides information on chemical entities with various physicochemical and pharmacokinetic properties, standardized biological activity data, systematic taxonomy and geographical distribution of source organisms, and detailed literature citations	https://www.cmnpd.org/ (accessed on 26 January 2024)	^1^
TimTec NPL	800	Tap natural bioactivity potential with molecules that are primarily sourced from plants with the remaining samples from bacteria, fungus, and animal sources.	http://www.timtec.net/ (accessed on 26 January 2024)	^1^
COCONUT(COlleCtion of Open Natural ProdUcTs)	407,270	One of the biggest and best annotated resources for NPs available free of charge and without any restriction.	https://coconut.naturalproducts.net/ (accessed on 26 January 2024)	^1^
TriForC	266	A pipeline for the discovery, sustainable production and commercial utilization of known and novel high-value triterpenes with new or superior biological activities	http://bioinformatics.psb.ugent.be/triforc/#/home (accessed on 26 January 2024)	^1^

^1^ Data is sourced from respective database websites.

## Data Availability

No new data were created or analyzed in this study. Data sharing is not applicable to this article.
